# Ventricular Assist Device-Specific Infections

**DOI:** 10.3390/jcm10030453

**Published:** 2021-01-25

**Authors:** Yue Qu, Anton Y. Peleg, David McGiffin

**Affiliations:** 1 Infection and Immunity Theme, Monash Biomedicine Discovery Institute, Department of Microbiology, Monash University, Clayton, VIC 3800, Australia; yue.qu@monash.edu; 2 Department of Infectious Diseases, The Alfred Hospital and Central Clinical School, Monash University, Melbourne, VIC 3004, Australia; 3 Department of Cardiothoracic Surgery, The Alfred Hospital, Monash University, Prahran, Melbourne, VIC 3004, Australia

**Keywords:** ventricular assist device, driveline infections, exit-site, driveline tunnel, biofilms, epidemiology, prevention, treatment

## Abstract

Ventricular assist device (VAD)-specific infections, in particular, driveline infections, are a concerning complication of VAD implantation that often results in significant morbidity and even mortality. The presence of a percutaneous driveline at the skin exit-site and in the subcutaneous tunnel allows biofilm formation and migration by many bacterial and fungal pathogens. Biofilm formation is an important microbial strategy, providing a shield against antimicrobial treatment and human immune responses; biofilm migration facilitates the extension of infection to deeper tissues such as the pump pocket and the bloodstream. Despite the introduction of multiple preventative strategies, driveline infections still occur with a high prevalence of ~10–20% per year and their treatment outcomes are frequently unsatisfactory. Clinical diagnosis, prevention and management of driveline infections are being targeted to specific microbial pathogens grown as biofilms at the driveline exit-site or in the driveline tunnel. The purpose of this review is to improve the understanding of VAD-specific infections, from basic “bench” knowledge to clinical “bedside” experience, with a specific focus on the role of biofilms in driveline infections.

## 1. Introduction

**Figure 1:**
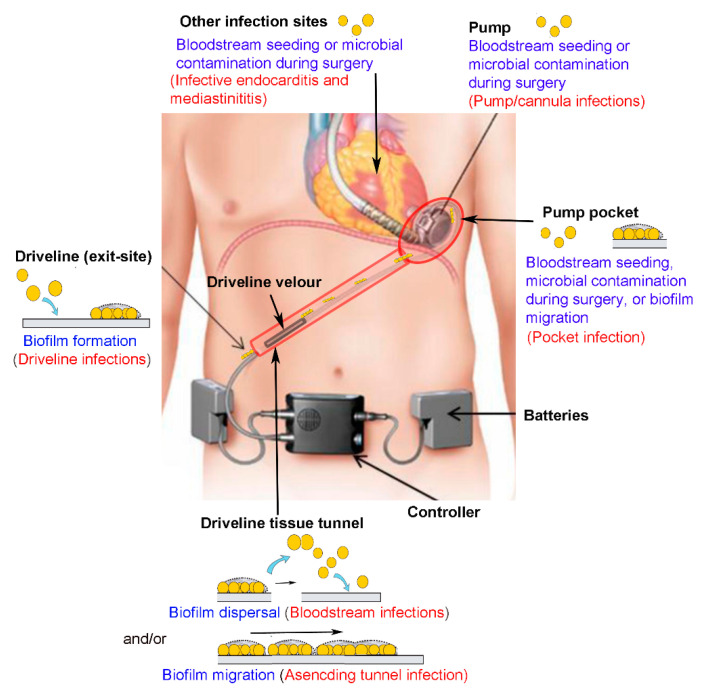
Ventricular assist device (VAD)-specific and VAD-related infections. Microorganisms cause infections (text in red) at different anatomic sites where VAD components are placed (text in black) via three routes (text in blue). Microorganisms form biofilms on the driveline at the skin exit-site, causing superficial driveline infections. Microbial dispersal from established biofilms often results in bloodstream infections. Migration of biofilms along the driveline tissue tunnel leads to ascending tunnel infection or pump pocket infection (route 1). Intraoperative microbial contamination of VAD components (route 2), or hematogenous seeding from other infection sites (route 3) can also lead to pump pocket infection, pump/cannula infections, or other VAD-related infections.

**Figure 2:**
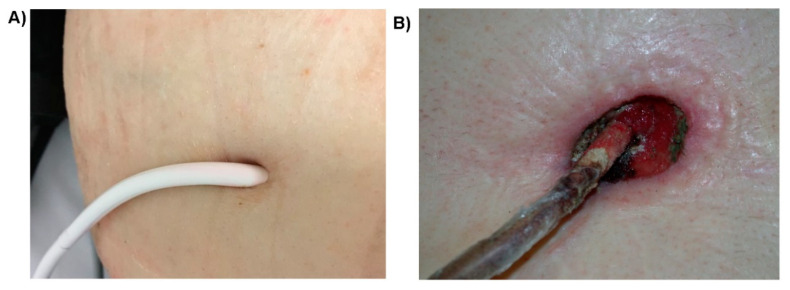
Driveline exit-site infection. (**A**) Uninfected control. The patient received a HeartMate II VAD system (Abbott, Plymouth, MN, USA). (**B**) A typical driveline exit-site infection that demonstrates tissue destruction and granulation tissue. A HeartWare HVAD system (Medtronic, Minneapolis, MN, USA) was used in the patient.

Heart failure is a growing public health issue affecting at least 26 million people worldwide [1]. Ventricular assist device (VAD), an electrically powered implantable rotary blood pump, has been used as an advanced treatment for heart failure, either as a “bridge to transplantation” for patients waiting for a donor heart, or as “destination therapy” for those who are ineligible for a heart transplant [2]. The driveline is a percutaneous tube connecting the internal VAD pump and the extracorporeal controller unit and batteries (Figure 1). A typical driveline consists of segments of smooth tubing made of polyurethane or silicone, and a proximal segment of tubing to which velour made of layers of Dacron polyester fibers is bonded. The percutaneous nature of the driveline and the presence of a skin-driveline interface renders patients highly susceptible to microbial contamination and infection. Driveline infections are known as the Achilles heel of VAD therapy since it potentially jeopardizes the benefits of VAD support [3] (Figure 2). These infections often start at the driveline exit-site causing local infection but may then extend along the driveline tissue tunnel to the pump pocket, or spread to the bloodstream, and hematogenously to distant sites [3,4,5,6,7] (Figure 1).

Biofilm formation is a major microbial strategy underpinning the pathogenesis and persistence of recalcitrant driveline infections [8,9]. This specific growth model of microbial pathogens not only initiates infection at the driveline skin exit-site, but also endows the infective process with an antimicrobial-resistant property and facilitates the extension of infection to deeper tissues [8,9]. Despite the introduction of newer generations of VADs and various medical and surgical infection prevention strategies, driveline infections still occur at a substantial prevalence of 10–20% annually [10,11]. It has been well recognized that the presence of a driveline infection does not necessarily disqualify such a patient proceeding to a heart transplant and does not negatively impact post-transplant survival [5,12], provided that the transplant is not performed in the setting of a sepsis syndrome [13]. However, driveline infections do affect VAD patients in many other ways, including impairing quality of life, contributing to other implant-related complications such as severe sepsis and hemorrhagic or ischemic stroke, and contributing to post-transplant complications such as wound infection and peri-operative bleeding [3,14,15,16]. The high economic burden associated with prolonged hospitalization or frequent hospital re-admission is now one of the main hurdles for the wide use of VADs for destination therapy.

This review will outline our current understanding of VAD-specific infections, in particular, their pathogenesis, epidemiology, diagnosis, prevention and treatment, and provides guidance on the clinical management of this important complication of VAD implantation.

## 2. Definition and Epidemiological Profile of VAD-Specific Infections

### 2.1. Classification of VAD-Associated Infections

The introduction of standardized definitions of infections in VAD patients by The Infectious Diseases Council of the International Society of Heart and Lung Transplant (ISHLT) allowed classification of infections related to VAD implantation. VAD-associated infections can be divided into three categories, VAD-specific, VAD-related and non-VAD infections [17,18]. VAD-specific infections refer to those directly related to a component of the VAD system, including the driveline, driveline tunnel, pump pocket, pump and the inflow or outflow cannula. VAD-specific infections may be introduced intra-operatively at the time of implant, or acquired via the driveline exit-site post-implantation, or, less frequently, from other infectious sources via hematogenous spread (Figure 1). VAD-specific infections can be further divided into superficial or deep infections. A superficial infection involves soft tissue outside the fascia and muscle layers, whereas deep infections span beyond these structures [18]. Driveline infections are the most frequently encountered VAD-specific infections, often involving soft tissues surrounding the driveline tubing and presenting erythema, warmth, and purulent discharge at the skin exit-site (Figure 2). VAD-related infections refer to infections that may be impacted by VAD implantation, including infective endocarditis (IE), bloodstream infections (BSI), and mediastinitis. VAD-related infections are not limited to VAD patients. The presence of a VAD, however, complicates their clinical diagnosis and management, and may increase their incidence in comparison with that in non-VAD patients. Pneumonia and urinary tract infections occurring in VAD patients are categorized as non-VAD infections as they are unrelated to the VAD [17,18]. Based on the time to the onset of infections, VAD-specific infections can be further grouped into early-onset (<1 month after implantation) and late-onset infections (>1 month after implantation) [3,19]. Early-onset infections are often due to the intraoperative or perioperative contamination of operative sites, whereas late-onset infections, especially those related to the driveline, are likely due to lack of proper care of the driveline exit-site.

### 2.2. Prevalence of VAD-Specific Infections

Advances in VAD design and manufacturing, surgical implant techniques, and VAD patient care program have significantly changed the epidemiological pattern of VAD-specific infections [20]. Replacement of pulsatile-flow (PF) VAD systems with newer continuous-flow (CF) VAD systems has led to significantly fewer pump and pocket infections, possibly due to the intra-pericardial location of the CF-VAD pump that avoids the large pre-peritoneal pocket needed for PF-VADs [21,22]. Driveline infections, however, are still frequently encountered in many VAD patients and remain the commonest VAD-specific infection.

Several large-scale, multicenter mechanical circulatory support (MCS) registries, including the North American based Interagency Registry for Mechanically Assisted Circulatory Support (INTERMACS), the European Registry for Patients with Mechanical Circulatory Support (EUROMACS), the Trans-Atlantic Registry on VAD and Transplant (TRAViATA) and the ISHLT Registry for Mechanically Assisted Circulatory Support (IMACS) have reported an incidence of 9–11.2% per patient-year for driveline infections [23,24,25,26,27]. Other large-scale non-registry studies also reported comparable prevalence of 10–20% for driveline infections in a 12-month period post-VAD-implantation [28,29,30]. Unlike other cardiovascular implantable electronic device (CIED) infections, the occurrence of driveline infections continues for the duration of the implant, with Sharma et al., (2012) reporting the odds of developing a driveline infection rising by 4% for every month of device support [31]. Stulak and associates also found time-related cumulative risks of 7%, 20%, 25% at 1, 3 and 5 years, respectively, for driveline infections [26].

Although the median time to all VAD-associated infections was reported to be 68 days [32], many recent studies using CF-VADs have found that driveline infections mostly present as late-onset infections, occurring between 2–6 months after implant [30,31,32,33,34,35]. Few cases actually develop within 1-month post-implantation [28,36]. Studies examining the readmission profile of patients with VADs have found that driveline infections often peak at 6-months post-implantation, when patients are more mobile and independent after hospital discharge [15,28,34].

### 2.3. Risk Factors of VAD-Specific Infections

Many risk factors for VAD-specific infections have been reported. Those that have been widely accepted are duration of support, repetitive exit-site trauma caused by shearing traction or torsion injury on the driveline, and large body mass index (BMI) and diabetes of the patient [3,27,34,36]. Other inconclusive risk factors include region of residence, younger age and related higher activity rates, older age and related patient morbidity, depression status of patients, renal dysfunction/elevated serum creatinine level, severe heart failure, malnutrition, T-cell dysfunction, blood product administration, hypogammaglobulinemia, the presence of intravascular lines, delayed sternal closure, prolonged operation, longer than usual intensive care and postoperative hospital stay, and location of driveline exit site [3,27,32,37].

## 3. Medical Significance of VAD-Specific Infections

### 3.1. VAD-Specific Infection, Heart Transplant, and Pre-Transplant and Post-Transplant Mortality

In general, VAD-specific infections, including driveline infections and pocket infection, have no direct impact on a successful heart transplant, and the survival before or after transplantation [12,38,39,40]. Although previous studies suggested that VAD patients who developed sepsis were less likely to be bridged to cardiac transplantation [39,41], it is now well established that under the coverage of appropriate antibiotics, heart transplantation can be successfully performed for VAD patients with VAD-specific infections [39,42]. In fact, VAD removal at heart transplant can be curative for recalcitrant driveline infections or VAD endocarditis.

Diminished survival to transplantation in VAD patients was found to be only related to the occurrence of VAD-related infections such as bloodstream infections and associated sepsis [6,28,32,34,39,43]. A recent large non-registry cohort study including 455 patients with CF-VADs found that patients with VAD-related infections, mostly BSI, had a shorter median survival than patients with VAD-specific infections [29]. Among VAD-related BSI, those caused by fungi have the highest hazard ratio, followed by that of Gram-negative and Gram-positive bacteria [44].

VAD-specific infections, in particular, driveline infections, however, may indirectly affect the pre-transplant and post-transplant outcomes of VAD patients because of an increased risk of developing into VAD-related BSI or cannula infections [32,45]. A VAD-related BSI was defined as one where the same pathogen was cultured from the device and the blood with no other obvious source [44]. It has been found that driveline infections often share a similar microbiological profile to VAD-related BSI [44]. Biofilms that have grown on drivelines in the tissue tunnel may serve as a nidus of infection for microorganisms circulating in the bloodstream [28]. In large-cohort studies, 30–50% of VAD-related BSI were found to be associated with driveline infections [32,44], with a trend showing higher cumulative incidence of BSI linked to deeper driveline infections [6]. An earlier study that analyzed isolates obtained from the bloodstream and infected drivelines using pulsed field gel electrophoresis (PFGE) also suggested that the driveline was a major portal of entry for nosocomial bloodstream infections in patients on VAD support [46].

Stroke is probably the most devastating neurological complication following VAD implantation, responsible for significant mortality and impairment of quality of life [5,13,47]. Multivariable analyses have recently identified an evident association between VAD-associated infections and both ischemic and hemorrhagic strokes [47,48]; hemorrhagic strokes occur more frequently than ischemic strokes in VAD patients with VAD-associated infections [49]. Interestingly, patients with VAD-related or non-VAD infections, but not VAD-specific infections have an increased risk of a stroke [36,43,49]. This is consistent with findings from a recent study that suggested less likelihood of stroke-related pathological changes such as cerebral microbleeds in patients with superficial driveline infections compared with patients with other infections [50].

### 3.2. Pre-Transplant Infective Status Often Predicts Post-Transplant Infections

Pre-transplant infection status is considered a risk factor that predicts persistent post-transplant infections in VAD patients after cardiac transplantation [39,51,52]. Post-transplant infections often occur in former driveline or pocket sites [39,51]; these infections do not appear to directly affect post-transplant survival [39]. There is no correlation between the causative pathogens in pre- and post-transplant infections, implying that the former infected driveline tunnel or pocket may create an environment favorable for microorganisms to seed and proliferate [51].

## 4. Microbiological Profile of VAD-Specific Infections

Despite the recent replacement of PF-VAD systems with the more advanced CF-VAD systems, and increased use of VADs for destination therapy, the microbiological profile of VAD-specific infections remains unchanged [30,32,33,44,53].

### 4.1. Bacterial, Fungal or Polymicrobial Origins

The most common pathogens isolated from VAD patients with confirmed driveline infections or pocket infection are Gram-positive bacteria that colonize skin and the nasal cavity, particularly *Staphylococcus aureus* and *Staphylococcus epidermidis* [54,55]. These two species cause at least half of all driveline infections [33,37,55]. Other Gram-positive bacteria frequently implicated in driveline infections include *Enterococcus* species, *Corynebacterium* spp., *Streptococcus pneumoniae* and non-epidermidis coagulase-negative staphylococci [31,32,33,37]. *Pseudomonas aeruginosa* is the leading Gram-negative bacterium accounting for approximately a quarter of all driveline infections [33,55]. Other Gram-negative pathogens often causing driveline infections include *Klebsiella pneumoniae*, *Acinetobacter baumannii*, *Enterobacter* spp. and *Serratia* spp. [30,31,32,33]. Etiological agents differ between VAD-specific infections of different onset time. *S. aureus*, *S. epidermidis*, and some other Gram-positive bacteria can be readily acquired at the implant hospitalization and are often involved in early-onset infections. Late-onset infections are more likely to involve *P. aeruginosa* and other Gram-negative bacteria, probably reflecting frequent contact of recovering patients after hospital discharge with highly humid home environments such as a shower without protecting the driveline exit-site [37,56].

The previously mentioned finding that the presence of a VAD specific infection does not necessarily compromise pre-transplant or post-transplant survival cannot be extended to fungal infections. Fungi are not common causes of driveline infections; these microorganisms are more frequently isolated from infections of deeper tissues such as the VAD pocket or the bloodstream [33] and are usually associated with an extremely poor outcome [57,58,59,60]. *Candida albicans* is the most common fungal pathogen causing VAD-specific infections, followed by *Candida glabrata*, *Candida kruseii* and *Candida parapsilosis* [55]. *Aspergillus* species have also been identified as a rare cause, with most information reported post-mortem [59]. When a VAD patient presents with clinical manifestations of sepsis, and a *Candida* species is isolated from the driveline site or the pump pocket but not the bloodstream, urgent transplantation has been recommended as the most appropriate management [60].

About 10–20% of VAD-specific infections are polymicrobial involving multiple microorganisms from the same or different kingdoms (bacterial polymicrobial infections or fungal-bacterial polymicrobial infections) [30,32]. Polymicrobial growth and interactions in a device-related infection can result in a more complex pathological process and a greater challenge for treatment [61].

### 4.2. Microbial Pathogenesis: The Important Role of Biofilm Formation in VAD-Specific Infections

**Figure 3:**
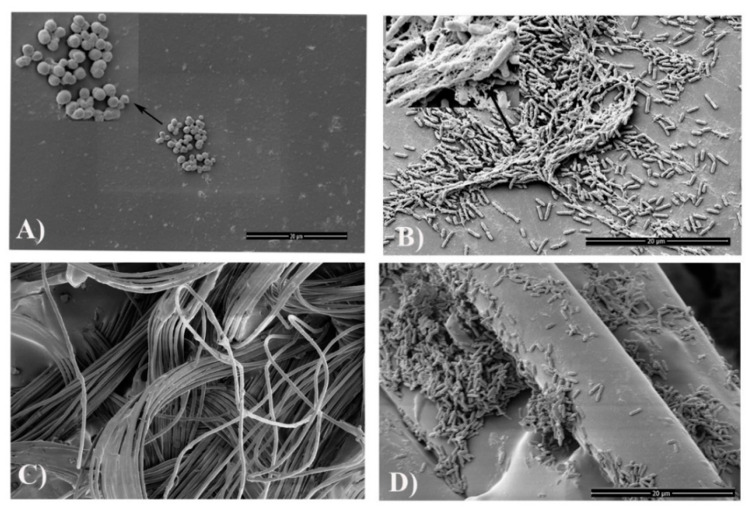
In vitro biofilm formation on different components of a VAD driveline [9]. (**A**) Biofilms formed by *S. aureus* ATCC 25923 on the smooth section of the driveline; (**B**) Biofilm formation of *P. aeruginosa* PAO1 on the smooth section of the driveline; (**C**) Three-dimensional structure of the driveline velour; (**D**) Biofilm formation of *P. aeruginosa* PAO1 on the velour section. Drip flow biofilm reactor assay was used to mimic the clinical environment where an implanted driveline and invading microorganisms might encounter. Reprinted from Reference [9], Copyright (2020), with permission from Elsevier.

**Figure 4:**
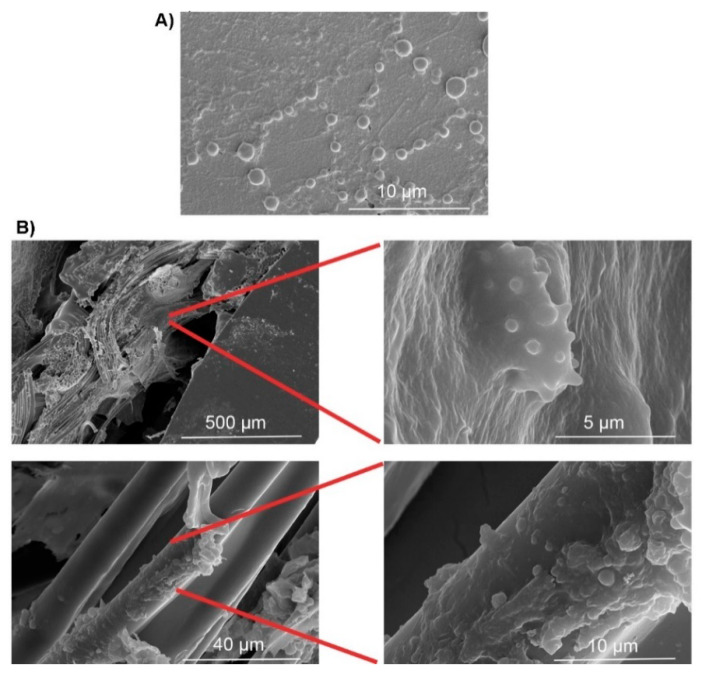
Clinical biofilms observed on an infected VAD driveline [8]. A driveline was explanted from a VAD patient with clinically diagnosed *S. aureus* driveline infection. The driveline was sectioned into small pieces, washed to remove planktonic cells, structurally fixed with glutaraldehyde, and imaged with scanning electron microscopy. (**A**) *S. aureus* monolayer biofilms formed on the smooth section of the driveline around the exit-site; (**B**) Microcolony-alike biofilms formed at the tissue-driveline interface (upper panels) or on the velour fibers (lower panels). Reprinted from Reference [8], Copyright (2020), with permission from Elsevier.

The key pathogenic mechanism of VAD-specific infections involves the interaction between the implanted VAD/driveline, the invading pathogens and host responses, which lead to the formation of microbial biofilms at the device-human tissue interface [8,9,54,62]. Biofilms are a self-protecting growth mode of microorganisms with densely-grown cells often embedded in extracellular polymeric substances (EPS); they display heterogeneity in growth rates and tolerance to external environmental stressors [63,64]. Once a microbial biofilm is established on the surface of biomaterials, the embedded microorganisms may become extremely difficult to eradicate by antimicrobials and the human immune system [65]. Early biofilm studies of VAD systems mostly focused on drivelines at the skin exit-site [54,66]. Opportunistic pathogens such as skin colonizers of the exit site, including *S. aureus* and *S. epidermidis*, or those often encountered in a humid domestic environment, such as *P. aeruginosa*, can readily form biofilms at the driveline exit site. Micro-trauma of the exit-site predisposes to bacterial invasion of the driveline and subcutaneous tunnel. The importance of drivelines in this process was highlighted in our recent in vitro study, which showed the predilection of different pathogens to different parts of the driveline and the importance of the subcutaneous tunnel as a key driver of recalcitrant driveline infections [9] (See Figure 3A,B,D). For example, we found that the scaffold provided by the three-dimensional structure of the driveline velour (Figure 3C,D) facilitated pathogens such as *P. aeruginosa* and *C. albicans* to form more robust biofilms [9]. The important role of biofilms in driveline infections was confirmed when our group characterized in vivo biofilms in VAD patients with clinically diagnosed driveline infections (Figure 4) [9]. Such biofilms have unique morphological characteristics, presenting as densely grown microbial clusters using either the driveline or human tissue as a supporting base. These small clusters exhibit similar traits as in vitro biofilms, including high resistance to conventional antimicrobial agents [8].

### 4.3. Microbial Route for VAD-Specific and VAD-Related Infections: The Important Role of Biofilm Migration

There are three main routes by which VAD components become colonized by microorganisms: contamination of VAD components or relevant surgical sites at the time of surgery; hematogenous seeding via bacteremia from other infection sites; alternatively, most commonly by direct deposition of microorganisms at the driveline exit-site and migration towards other VAD components (Figure 1).

**Figure 5:**
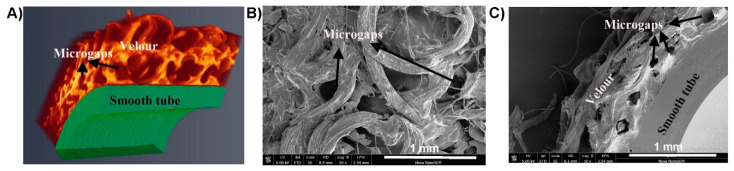
The presence of microgaps in the driveline tissue tunnel. (**A**) Micro-CT image of an explanted driveline shows numerous microgaps in the sectioned velour; (**B**) Scanning electron microscopy (SEM) of the velour (top-bottom view) shows microgaps [9]; (**C**) SEM of the explanted driveline (cross-section view) also shows numerous microgaps within the velour, suggesting insufficient tissue integration [9]. Reprinted from Reference [9], Copyright (2020), with permission from Elsevier.

Migration of microbial biofilms in VAD-specific infections can facilitate the extension of superficial driveline exit-site infections to deeper tissues and cause more severe tunnel, pocket, pump, or bloodstream infections. Retrospective studies by others found that most driveline infections started superficially, and their depth progressed to involve the bloodstream, deep sections of drivelines, or even the pump pocket over months on VAD support, suggesting biofilm migration might play a critical role in the infection spread [6,38]. Release of planktonic cells from a mature biofilm formed on the driveline can result in seeding of neighboring tissues with pathogenic microorganisms and more importantly, hematogenous dissemination of microorganisms to remote tissues where the other components of VAD are placed [62]. In addition to the “indirect” bloodstream pathway, direct migration of biofilms along the driveline tunnel may also enable pathogens to access deep-tissues, resulting in severe infections [9,54,67]. Toba et al., used an in vivo mouse model and demonstrated that biofilm expansion enabled bacteria to “migrate” along the driveline [54]. We also found that clinical biofilms often extended from the exit-site to deeper tissue tunnels [8]. Different microbial species differed in their ability to migrate along the driveline and this might explain the change in the dominant microorganisms causing VAD-specific infections of different depths [54]. Clinically, host tissue integration of the driveline velour has been used to stabilize the driveline in the subcutaneous tissue tunnel and to prevent late-onset infection [10]. We have observed numerous microgaps in the velour section of implanted drivelines, suggesting that insufficient tissue integration has been achieved (Figure 5). These microgaps may serve as a “conduit” that facilitate the migration of microbial biofilms to deeper tissues [8].

## 5. Diagnosis of VAD-Specific Infections

VAD-specific infections are often clinically diagnosed as “proven, probable or possible” infections. The “probable” or “possible” diagnoses are made based on clinical assessment, while the diagnosis for a “proven infection” requires a matrix with additional microbiologic and/or radiographic evidence.

### 5.1. Clinical Evidence

Typical clinical signs of driveline infections include purulent drainage from the exit-site, discoloration/induration and excessive erythema of the surrounding tissue, pain of local tissues including exit-site and driveline tunnel, and abscess formation [34]. Based on clinical presentation and disease severity, the Sharp Memorial group categorized driveline infections into 5 stages, including local healing disorder (stage 1), local infection (stage 2), systemic infection (stage 3), systemic infection with high severity (stage 4), and progressing systemic infection with deep driveline infection or ascending infection (stage 5) [68]. Recently, the Driveline Expert STagINg and carE DESTINE study group refined this classification by adding asymptomatic stage 0, and subdividing stage 0, 1 and 2 into two sub-stages respectively to facilitate early recognition of driveline infections [69].

### 5.2. Microbiological Evidence

In the context of a clinical exit site infection, a microbiological swab can be readily obtained and a positive culture may identify the pathogen(s) causing the exit-site infection. Isolation and identification of pathogens from beyond the driveline exit-site remains a clinical challenge. Gordon et al., (2013) reported that driveline infections often involved both the exit-site and deeper tissues such as the tunnel, the pocket and even the pump [32]. Invasive exploration by intraoperative sampling or needle aspiration under the guidance of ultrasound or computed tomography (CT) is possible but undesirable [70] owing to the risk of damaging VAD components and introducing new microorganisms [71]. Our recent study examining clinical drivelines explanted from VAD patients with confirmed driveline infections caused by *S. aureus*, *P. aeruginosa*, *S. epidermidis*, *Corynebacterium jeikeim*, *Sphingomonas parapaucimobilis* or mixed microorganisms respectively found that driveline tunnel infection often co-existed with exit-site infection [8]. Both HeartMate 3 (Abbott, Plymouth, MN, USA) and HeartWare HAVD systems were used in our patients. Microorganisms cultured from the tunnel matched that isolated from the exit-site, suggesting that a swab of the exit-site, if showing a positive culture, can be useful for the prediction of microorganisms causing deeper tunnel infections [8]. It should be borne in mind that clinical specimens from the exit-site may be contaminated with skin flora and definitions of true infection always need to have clinical evidence of inflammation/infection.

### 5.3. Radiographic Investigations

Radiographic investigations can be carried out when infections of the driveline tunnel or the pump pocket is suspected. Although an ISHLT consensus document has recommended CT or ultrasound imaging to confirm or exclude deep driveline or other VAD-specific infections, by detecting fluid collections around VAD components [72], these conventional radiographic technologies have shown suboptimal sensitivities and specificities [29]. Nuclear radiologic modalities such as 18F-fluorodeoxyglucose positron emission tomographic imaging combined with CT (18F-FDG PET/CT) and 67gallium (67Ga) and 111indium (11In)-labelled leukocyte single-photon emission computed tomography-CT (SPECT/CT), have demonstrated great potential for quantifying the extent of deep VAD-specific infections [71,73,74,75]. Among nuclear radiologic modalities, 18F-FDG PET/CT appeared to be superior to leucocyte scintigraphy-based technologies in the context of general sensitivity and specificity, due to its high spatial resolution [75,76]. Two recent systematic reviews and meta-analyses assessed the performance of 18F-FDG PET/CT in diagnosing VAD-related infections, and both reported high accuracy of this dual-modality imaging system, supported by pooled sensitivities of 92% and 95%, and specificities of 83% and 91%, respectively [77,78]. Caution, however, should still be taken when using 18F-FDG PET/CT to detect biofilm-related driveline infections. The presence of EPS may allow biofilm-grown microorganisms to escape local immune and inflammatory responses and thus the detection by the PET/CT scan [73,79]. Post-operative inflammation and pathological accumulation of FDG on the velour may also confound the specificity of 18F-FDG PET/CT in detecting early-onset infections [80]; leucocyte scintigraphy has been recommended as a better option [75]. Other general limitations of nuclear radiologic modalities that need to be further addressed include insufficient clinical experience and lack of criteria for interpretation [30,80].

## 6. Prevention of VAD-Specific Infections

The trend in using VADs for long-term bridge to transplant therapy or destination therapy emphasizes the importance of prevention of VAD-specific infections [5].

### 6.1. Advances in VAD Design and Manufacturing

Advances in VAD systems have seen a significant decline in complications including VAD-associated infections [2]. The transition from the paracorporeal PF-VAD to the implantable CF-VAD has led to a significant decrease in driveline infections [33,81]. This might be due to less intrathoracic dissection required for CF-VAD implantation compared to that for PF-VADs, and the smaller diameter and higher flexibility of CF-VAD drivelines [3].

The incidence of driveline infection also differs among patients with different CF-VADs. The recent randomized controlled trial of MOMENTUM 3 found no difference in major infectious complications including driveline infections between the newer centrifugal-flow HeartMate 3 VAD and the older axial-flow HeartMate II (HMII) VAD, suggesting that the pump type is not a determinant factor of VAD-specific infections [82]. Controversial results have been reported when VADs from different manufacturers, such as HeartWare HVAD (Medtronic, MA, USA) and HeartMate VADs (St. Jude, Pleasanton, CA, USA) were compared. Two very recent retrospective studies found that patients with HeartMate 3 VAD were less likely to develop driveline infections or VAD-specific infections than those with HeartWare HVAD [83,84]. HeartMate II VAD and HeartWare HVAD were parallel in developing driveline infections in the studies by Haglund et al., (2015) and Stulak et al., (2016) [15,26]; other studies reported a lower incidence of driveline infections in patients with a HeartWare HVAD relative to that with a HeartMate II VAD [36,85,86]. The difference in the incidence of driveline infections between VADs from different manufacturers is possibly due to their differences in driveline flexibility, cable diameters and other driveline characteristics such as biomaterials used for driveline manufacturing [36,87]. Smaller outer diameter and lower stiffness of drivelines have been associated with less driveline infections [87]. Though there is a lack of information in the literature comparing different materials of the drivelines as predictors of driveline infections, studies on other implantable medical devices such as central venous catheters found that the nature of the biomaterials was one of the key determinants of device-related infections [88,89,90]. In general, microorganisms have a preference for adhering to silicone-based polymeric materials (HeartMate II or HeartMate III) or polyvinyl chloride (PVC), compared to that of Teflon or polyurethane (HeartWare HVAD) [9,88,89]. In addition to choosing a specific biomaterial, prevention of driveline infections may be achieved by engineering the driveline surface using a broad range of strategies, such as bonding the smooth tube with layers of velour to enhance tissue integration, or pre-treating the driveline surface with anti-infective coatings; the former has been adopted by many VAD manufacturers and the latter is still at the experimental stage.

### 6.2. Driveline Care and Patient Education

One important preventative strategy is to avoid micro-trauma of the driveline exit site, the fragile port of microbial entry and to minimize the risk of microbial contamination at the post-implantation stage. In general, hand and skin disinfection, exit-site preparation, using maximal sterile barrier precautions, and limiting the number of driveline manipulations are all critical in the prevention of driveline infections [91,92]. At the Alfred Hospital, Melbourne and many other VAD centres in the world, driveline care protocols usually involve daily or 2nd daily cleaning with 4% chlorhexidine followed by coverage of the exit-site by a self-adhesive dressing. Various dressings have been investigated, aiming for inhibition of microbial growth around the exit-site or promotion of tissue regrowth of the exit-site [24,93]. No compelling evidence from large-scale randomized studies has been provided to support the efficacy of using antimicrobial dressings. Instead, caution has been raised regarding using antimicrobial dressings due to concerns of induction of antimicrobial resistance [94]. A more convincing intervention with solid supporting evidence is to use anchoring devices to immobilize drivelines at the exit-site [6]. This strategy has been found to significantly minimize the risk of repeated micro-trauma at the exit-site and subsequent driveline infections [24,95,96]. Until very recently, the Central Europe-based Driveline Expert STagINg and carE (DESTINE) study group proposed the first standard of a care protocol [69]. This expert consensus provided a detailed standard operating procedure (SOP) for appropriate driveline exit-site care, with emphasis on essentials such as sterile dressing change, driveline immobilization, and an advanced wound staging approach for early recognition of driveline infections [69]. Worldwide, driveline care is still institution-specific due to different local realities involved in caring for these patients.

Patient and caregiver education by VAD coordinators and strict compliance to the standard care procedures are also key factors determining the occurrence of driveline infections after discharge from the hospital [91,92]. Patient education mainly focuses on driveline trauma prevention, sterile technique for home dressing of driveline exit-site, and other daily activities related to exit-site care such as showering and driving [97]. Less careful handling of drivelines by patients, or increased stress on the driveline as recovering VAD patients become more active can both result in a higher incidence of driveline infections [98]. Most VAD programs allow showering one month after VAD implant, provided that the exit-site is covered by a waterproof dressing [10]; some programs even allow patients to shower without a dressing, if antibacterial soap can be used and a sterile dressing can be immediately applied after shower [99]. There is some evidence that the incidence of *Pseudomonas* driveline infection can be lowered by preventing the exit-site from contacting humid or moist environments [100].

### 6.3. Antimicrobial Prophylaxis

Using antimicrobial prophylaxis to prevent VAD-associated infections, systemically and/or topically, remains a routine practice in many VAD centres [17,101]. The regimen for antimicrobial prophylaxis varies among different institutions and mostly relies on the centre’s experience and preference [17,37,102]. In the era of PF-VADs, many centres used two to four drug regimens, often including vancomycin, a cephalosporin, a quinolone, rifampicin, and fluconazole, intending to cover Gram-positive bacteria, Gram-negative bacteria, and fungi [17,101,102]. Most centres used intravenous administration of antimicrobials for 24 or 48 h post implantation [102,103]. Longer antimicrobial prophylaxis did not demonstrate superior effectiveness [104], while risking the development of antibiotic resistance and antibiotic-related clostridial infections.

Systemic prophylactic antimicrobial strategies have been significantly simplified since the arrival of CF-VADs. Many centres follow general cardiac surgery prophylaxis guidelines that recommend a cephalosporin (cefazolin or cefuroxime) for 24–48 h, which can provide sufficient coverage for both Gram-positive and Gram-negative bacteria [72,103,105,106,107,108]. The effectiveness of this practice has been supported by clinical evidence from large-scale retrospective studies [103,109]. First or second generation cephalosporins have been recommended; the second generation was preferred as it has a broader coverage of Gram-negative bacteria and a lower risk in inducing *Clostridium difficile* colitis [106,109]. It has also been recommended that systemic prophylactic antimicrobial strategies should be tailored to institution-specific pathogen prevalence and local susceptibility profiles [72,105]. For example, vancomycin can be given to VAD patients hospitalized in centres where methicillin-resistant *S. aureus* (MRSA) has been frequently isolated, often in combination with an aminoglycoside [106]. Routine antifungal prophylaxis is not recommended [59]. Regarding the timing of antibiotic initiation, administration of drugs within 60 min of the skin incision has been recommended, with additional dosing during surgery every 3 to 4 h if the short-half-life cefazolin is used [106]. It should be noted that effectiveness of systemic antibiotics in preventing percutaneous driveline infections has been questioned [104].

Topical or local antimicrobial prophylaxis has also been employed to prevent VAD-associated infections. A recent prospective study using whole genome sequencing found concordant genomes between *S. aureus* at baseline and that causing late driveline infections, supporting a link between *S. aureus* colonization and the occurrence of *S. aureus* infections [110]. This has also rationalized the use of mupirocin or chlorhexidine washes in many centres to reduce nasal or skin colonization prior to VAD implantation [56,101,102]. In many Australian hospitals, Medihoney Antibacterial Wound Gel is routinely used at the skin exit-site of VAD drivelines to prevent infections. Our recent in vitro study however, found suboptimal effectiveness of Medihoney Antibacterial Wound Gel as a prophylactic agent against driveline infections due to biofilm formation at the driveline exit-site [111]. Large scale, prospective and randomized studies are needed to examine the effectiveness of topical or local antimicrobial prophylaxis against VAD-specific infections.

### 6.4. Surgical Prevention Strategies

Surgical strategies have been introduced to reduce the incidence of driveline infections. Strict aseptic technique is essential for the preparation and testing of VADs in the operative field. Another widely accepted surgical practice that may lower the risk of infections is to anchor the driveline to the skin at the exit-site by a suture that is left for two weeks upon completing the implantation. This is to avoid a driveline traction injury that may disrupt the tissue integration within the driveline tunnel. A purse-string suture is also placed in the subdermal layer immediately at the exit-site to encourage sealing of the skin to prevent access of external microorganisms into the driveline tunnel. Driveline infections have also been reduced by not allowing any of the velour section to project externally beyond the exit-site [112,113,114]. To do that, the velour—smooth tube interface should be placed 2 to 3 cm from the exit-site within the subcutaneous layer [95,113]. The exact anti-infective mechanisms of this surgical strategy remain unknown but is likely associated with reduction of the risk of trauma-related injuries at the exit site, less dermal inflammation and faster skin integration [112,114]. A longer subcutaneous smooth section should be avoided as it might become an infectious nidus and facilitate the extension of infection to deep tissues [115]. Other surgical strategies that might combat VAD-specific infections include using a rectus-sparing technique to prevent ascending driveline infections [116], using a more horizontal as opposed to vertical pathway for tunneling to improve stability of drivelines and reduce traction injury [117,118], or using a double-tunnel technique (driveline tunneled into the fascia of the rectus abdominis muscle in the umbilical direction followed by a subcutaneous pathway) with a left-sided exit-site for better resistance against ascending infections [19,119,120]. Solid clinical data is still needed to support the anti-infective efficacy of the above-mentioned strategies [19,116,117].

## 7. Treatment of VAD-Specific Infections

An ISHLT consensus document that highlights information essential for the formulation of a treatment strategy for VAD-associated infections has been developed based on expert opinions and case-based data [72]. The information includes (1) identification of the causative pathogens; (2) clarification of infection location (pump/cannula, pocket, or drivelines), (3) the infection type (infective endocarditis, bloodstream infection, or mediastinitis), and (4) the transplant candidacy status (bridge to transplantation or destination therapy).

The current review focuses on VAD-specific infections, in particular, driveline infections. Treatment of driveline infections follows the principles recommended by ISHLT and follows an escalation scheme according to its severity: local redness can be treated by regular wound dressings and mechanical lavage, as well as oral antibiotics based on drug susceptibility testing results; if oral therapy is not successful, the patient should be hospitalized and intravenous antibiotics will be administered; surgical excision of the infected area, in combination with negative-pressure vacuum therapy is recommended for those who don’t respond to intravenous antibiotics; if this option fails, the patient should be placed on the high urgency list for transplantation; prompt device replacement or transplant is only considered if ascending driveline infections are confirmed.

### 7.1. Treatment of Uncomplicated Superficial Driveline Infections

Early intervention of superficial driveline infections potentially lowers the risk of progression to deeper infections. Although some low-grade infections of the driveline or the tunnel have been treated with local wound care [31,44], antimicrobial therapy is still an essential part of successful management. Superficial driveline infections without evidence of systemic illness or bloodstream infections may be treated with either empiric or microorganism-specific antibiotics [45]; ISHLT consensus recommends oral or intravenous antibiotics for a minimum of 2 weeks [72]. Topical antimicrobial strategies, such as using crystal violet solbase and cold atmospheric plasma topical treatment, and negative pressure wound therapy have also been investigated for superficial driveline infections and some success has been achieved [121,122]. Clinical efficacy of these strategies, however, was based on studies of very small sample sizes and will need further validation by large-scale clinical trials [121,122].

### 7.2. Treatment of Deep VAD-Specific Infections

Treatment of deep VAD-specific infections is more challenging and often requires an aggressive treatment algorithm [3]. The Sharp Memorial Group suggests that patients with driveline infections may need to be hospitalized for intravenous antibiotic therapy if systemic symptoms appear [68]. ISHLT consensus also suggested 6–8 weeks of intravenous antibiotics for deep driveline or pocket infections, or driveline infections of uncertain depth, followed by long-term oral antibiotic suppression therapy targeting the causative pathogen/s [72]. Ekkelenkamp et al., recently analyzed treatment outcomes of VAD patients infected by *S. aureus* and found infections often relapsed after long-term intravenous or oral antibiotics were discontinued [45]. These relapses are most likely due to failure of antibiotics to eradicate biofilm-grown microorganisms causing deep driveline infections. Biofilm-active antimicrobial agents might be a better option for deep VAD-specific infections [123]. Rifampicin is a typical biofilm-active agent and has been found to be effective in treating deep driveline infections caused by *S. aureus* and *S. epidermidis* [123]. Caution should be taken when using rifampicin systemically due to its interaction with warfarin anticoagulation therapy, which is essential for VAD patients, potentially leading to life-threatening pump thrombosis or stroke [72].

Surgical intervention is another important measure for the management of deep VAD-specific infections [30,44,72,115,124]. Driveline infections of stage 4 and 5, as classified by The Sharp Memorial Group, when the infection is tracking down the driveline tunnel, often requires more aggressive surgical treatment, such as opening and draining the tunnel, debridement, re-tunneling of the infected driveline, and vacuum-assisted closure to facilitate tunnel healing [31,68,125]. Omentum may also be useful for wrapping and salvaging an infected driveline or a pump [126]. Local antibiotics have emerged as a promising complementary therapy for surgical treatment of recalcitrant and ascending driveline infections. Antibiotic-impregnated beads may be placed along an infected driveline tunnel or in the pump pocket [95,127,128], and allow slow elution of antibiotics into the surrounding tissue to eradicate microbial biofilms [128,129]. However, concerns have been raised regarding the in vivo efficacy, toxicity and hypersensitivity of locally using antibiotic beads. In addition, antibiotic beads are a vehicle to deliver high concentrations of antibiotics to infected tissue; the choice of antibiotics should be rationalized based on microorganisms isolated from the infection site, their antimicrobial sensitivity pattern, and the growth mode of the invading pathogens. Gentamicin, vancomycin, and tobramycin have been frequently used in antibiotic bead therapy; gentamicin and vancomycin were found to be ineffective against mature biofilms formed by *S. aureus* and *S. epidermidis* [129,130].

For VAD patients with more severe infectious complications such as relapsing pump bacterial infections, or fungal driveline infections, removing the pump and cardiac transplantation for bridge to transplant patients or exchanging the pump for destination therapy patients might be necessary. Before a suitable donor can be found for VAD recipients who have developed a fungal driveline or pocket infection, targeted antifungal therapy should be given [60]. Unlike other medical device-related infections, exchange of VADs for source control is generally avoided due to the complexity of surgical procedures and concerns regarding the risk of reinfections of newly implanted devices [10]; removal of VADs for a heart transplant is a preferred clinical strategy for dealing with severe VAD-associated infections, in particular those associated with complications. Moazami et al., (2013) in their multicenter study reported that only 0.6% of patients with a HeartMate II VAD underwent pump exchange due to device-related infections [131]. Yost and colleagues (2020), however, recently suggested reserving VAD exchange as a critical clinical strategy, as this procedure could still be carried out safely and effectively for patients with ongoing VAD-related or VAD-specific infections, with long-term outcomes comparable to primary VAD implantation [132]. It should be noted that over half of the patients in the study by Yost et al., (2020) experienced recurrence of infections in a 12-month period after device exchange [132].

## 8. Conclusions and Prospective: Rethinking of Prevention and Treatment of Driveline Infections Based on the Biofilm-Growth Mode of Invading Microorganisms

Introduction of transcutaneous energy transfer systems (TETS) will eliminate the need for a driveline and therefore driveline infections [133]. Until the TETS system becomes reliable for clinical use, driveline infections will continue to be a challenge for long-term VAD success. Biofilm formation and migration remain a significant factor that not only contributes to the establishment of VAD-specific infections, but influence their prevention and treatment.

Multiple strategies have been proposed to reduce the incidence of VAD-specific infections, including those commercialized by VAD manufacturers, those introduced peri-operatively, intraoperatively and post-operatively by medical practitioners (exit-site care, patient education, antimicrobial prophylaxis, and optimized surgical techniques), and those still at an experimental stage such as designing drivelines with improved skin/tissue integration and equipping device surfaces with antimicrobial coatings [134]. Driveline infections still occur at a substantial rate and continue to be an important source of morbidity for VAD patients. Developing more effective prevention strategies for VAD driveline infections, based on a comprehensive understanding of the role of pathogenic biofilms in driveline infections, is urgently needed. Our findings of microgaps in the driveline tissue tunnel suggested that further enhancing tissue integration of the implanted driveline might be a solution for late-onset driveline infections, by hindering biofilm formation and migration [28]. Similarly, treatment of recalcitrant driveline infections should be tailored more specifically to target biofilms grown at the interface of the driveline and tissue tunnel or within the velour structure. Such biofilms are difficult to eradicate by either systemic antibiotics or surgical interventions. Local use of biofilm-active antibiotics at high concentration for an extended period may provide hope in curing deep VAD-specific infections, but further research into optimized biofilm acting antimicrobials is desperately needed [64].

## Data Availability

Not requried for a review article.
